# Image-guided surgery and craniofacial applications: mastering the unseen

**DOI:** 10.1186/s40902-015-0037-x

**Published:** 2015-11-21

**Authors:** James C. Wang, Laszlo Nagy, Joshua C. Demke

**Affiliations:** 1 School of Medicine, Texas Tech University Health Sciences Center, Lubbock, TX USA; 2 Department of Surgery, Texas Tech University Health Sciences Center, Lubbock, TX USA; 3 Pediatric Neurosurgery, Department of Pediatrics, Texas Tech University Health Sciences Center, Lubbock, TX USA; 4 Facial Plastic and Reconstructive Surgery, Department of Otolaryngology, Texas Tech University Health Sciences Center, 3601 4th Street STOP 8312, Lubbock, TX 79430 USA

## Abstract

Image-guided surgery potentially enhances intraoperative safety and outcomes in a variety of craniomaxillofacial procedures. We explore the efficiency of one intraoperative navigation system in a single complex craniofacial case, review the initial and recurring costs, and estimate the added cost (e.g., additional setup time, registration). We discuss the potential challenges and benefits of utilizing image-guided surgery in our specific case and its benefits in terms of educational and teaching purposes and compare this with traditional osteotomies that rely on a surgeon’s thorough understanding of anatomy coupled with tactile feedback to blindly guide the osteotome during surgery. A 13-year-old presented with untreated syndromic multi-suture synostosis, brachycephaly, severe exorbitism, and midface hypoplasia. For now, initial costs are high, recurring costs are relatively low, and there are perceived benefits of imaged-guided surgery as an excellent teaching tool for visualizing difficult and often unseen anatomy through computerized software and multi-planar real-time images.

## Background

Craniofacial surgery can be complex, frequently requiring multiple surgeries over a patient’s lifetime. Thorough knowledge of craniofacial skeletal and soft tissue anatomy is necessary when planning and executing such complicated surgery, especially in cases involving syndromic craniosynostosis where severe aesthetic and functional deformities of the skull, orbits, and face exist. Image-guided surgery (IGS) uses preoperative imaging studies that are then viewed in multi-planar three-dimensional images intraoperatively allowing the surgeon to correlate physical landmarks with this radiographic map. IGS has known initial and recurring costs including increased setup time, registration, and calibration of instrumentation, which increases overall time under anesthesia.

## Methods

**Fig. 1:**
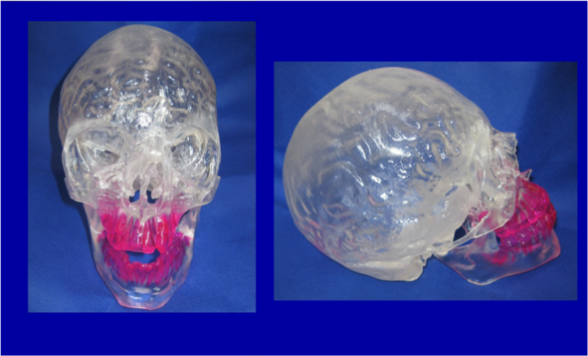
Frontal and right lateral view of stereolithographic model

**Fig. 2:**
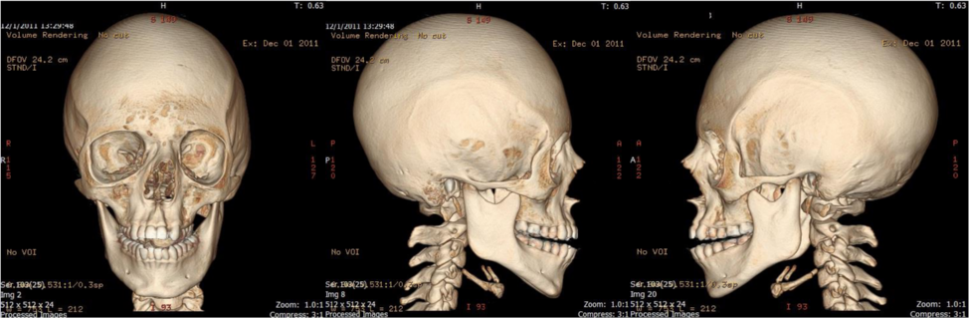
Preoperative frontal, right lateral, and left lateral three-dimensional computed tomography (CT) scans

**Fig. 3:**
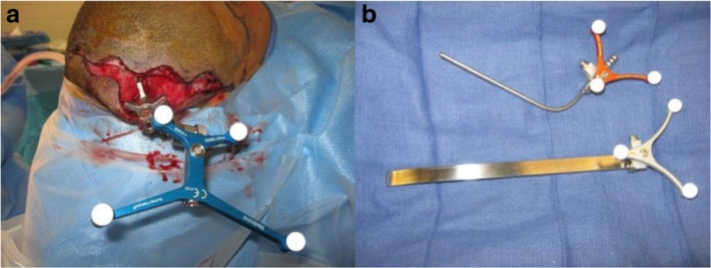
Intraoperative instruments. **a** A four-pointed head star with infrared spheres secured via intraoperative transcranial fiducial screw and (**b**) osteotome and suction with image-guided infrared optical spheres secured for registration

We utilized a stereolithographic model but did not do a virtual surgical plan (VSP) in a large part because the goal was to advance the orbits and mid face Leforte III to the point that the lagophthalmos and exorbitism was improved (Fig. 1). The StealthStation S7 IGS system using AxiEM tracking technology (Medtronic Sofamor Danek, Memphis, TN) is utilized by many surgical specialties [1–3]. It incorporates a recent preoperative scan, which is then registered with the patient in the operating room (OR) (Fig. 2). We scanned the patient’s face in the OR with a handheld laser to complete registration, and accuracy was verified by comparing the tip of the probe on the patient relative to the virtual probe on the IGS computer screen’s multi-planar images. Osteotomes and suction cannulas were registered as well (Fig. 3b) allowing these instruments to also be tracked during surgery.

**Fig. 4:**
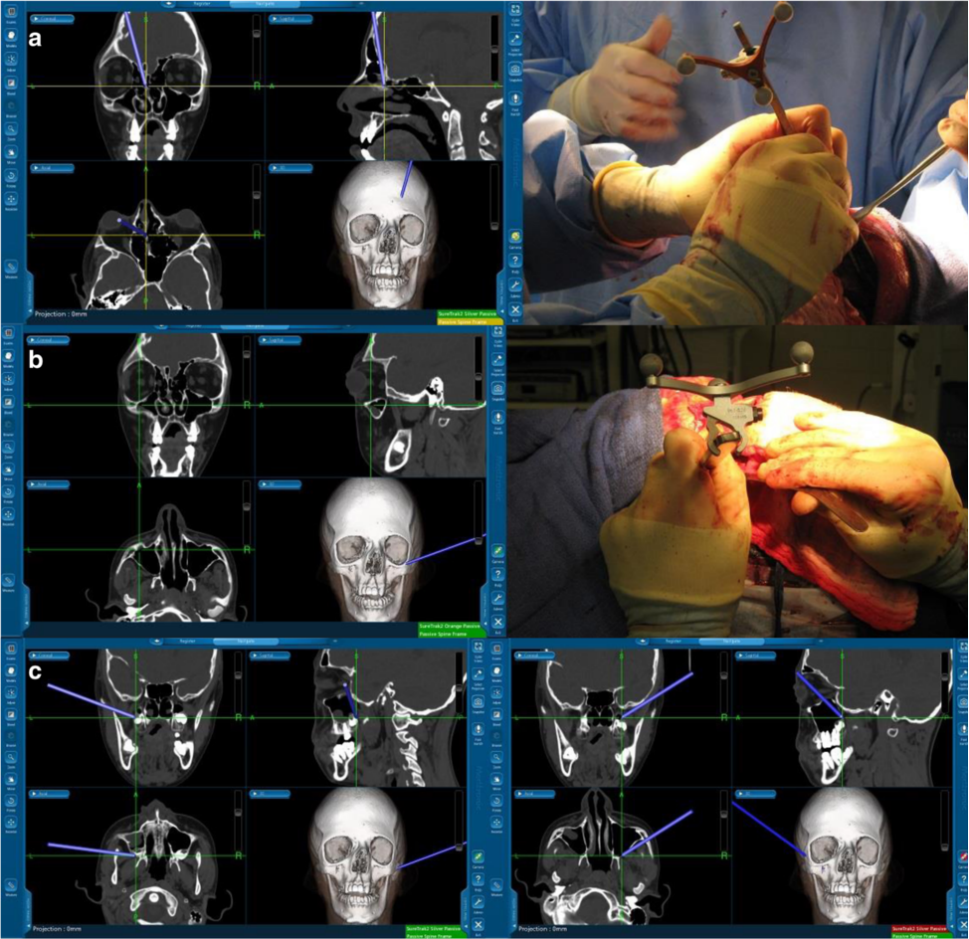
IGS navigation using Medtronic S7 and (**a**) registered osteotome behind right zygoma, **b** registered osteotome behind left zygoma, and (**c**) tip of registered osteotome in left pterygomaxillary fissure (*left*) and right pterygomaxillary fissure (*right*)

A custom orthodontic palatal splint was cemented and wired to the maxillary molars. A wavy-line bicoronal incision was marked and injected with local anesthetic. The scalp flaps were raised subgaleally; then separate pericranial and temporalis flaps were raised. A fiducial star with infrared spheres was screwed into the right parietal region (Fig. 3a). Dissection deep to the superficial layer of the temporal fascia was performed to protect the frontal branch of the facial nerve. Frontal craniotomy was performed, the bone flap was removed, and the orbital periosteum was freed up 360° around the eyes. The frontal sinus was cranialized. The dura was freed from the orbits and anterior cranial vault, and LeFort III osteotomies were performed from above with the aid of IGS registered osteotomes to track our position relative to the patient’s anatomic landmarks (Fig. 4). The additional time in setup and recalibration of the IGS system in this case was 30 additional minutes.

## Case presentation

**Fig. 5:**
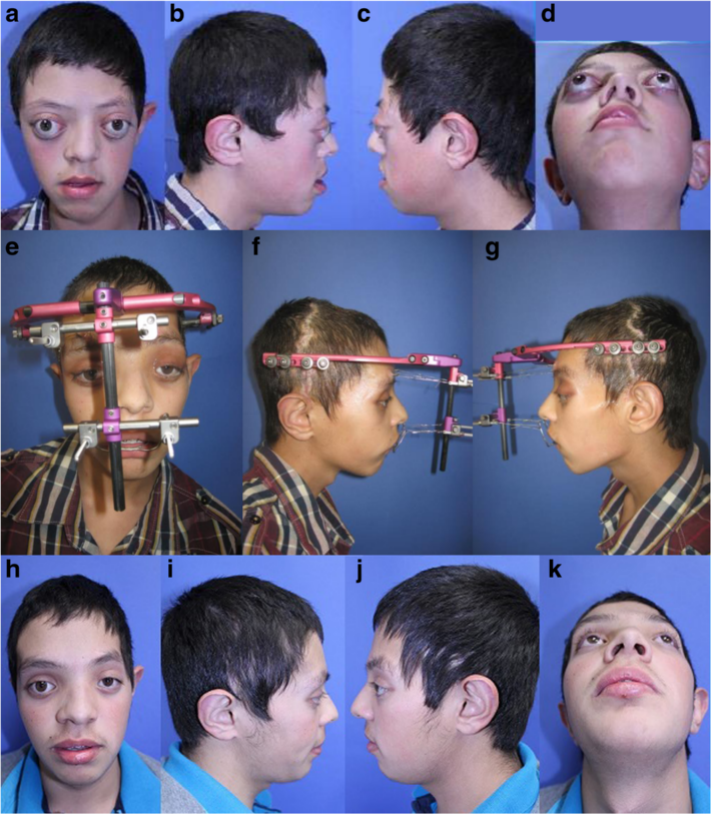
Photos of (**a**) preoperative frontal view, **b** preoperative right lateral view, **c** preoperative left lateral view, **d** preoperative basal view, **e** 2-month postoperative frontal view with REDII distractor, **f** 2-month postoperative right lateral view with REDII distractor, **g** 2-month postoperative left lateral view with REDII distractor, **h** 20-month postoperative frontal view, **i** 20-month postoperative right lateral view, **j** 20-month postoperative left lateral view, and (**k**) 20-month postoperative basal view

We present a 13-year-old boy with Crouzons and multi-suture synostosis with resultant brachycephaly, severe exorbitism, proptosis, malocclusion, midface, and maxillary hypoplasia (Fig. 5a–d). The frontal bone was secured to the supraorbital rim. The pericranial, temporalis, and scalp flaps were closed, frontal percutaneous screws were placed, and REDII (KLS Martin, Jacksonville, FL) halo head-frame was attached to the skull with finger-tightened percutaneous screws. After a 1-week latency period, 24-gauge wires were attached to the REDII, frontal screws, and the palatal splint. We began distraction osteogenesis (DO) at 0.75 mm twice a day for about 3 weeks to advance the midface, orbits, and frontal skull with goals to increase intracranial volume and orbital volume as well as improve facial aesthetics (Fig. 5e–g). Postoperatively, the patient stayed 12 days in the pediatric intensive care unit and stayed another week on the pediatric floor. We allowed another 3 months for bone consolidation with REDII removal in the office. The patient made exceptional progress and was followed regularly postoperatively (Fig. 5h–k).

## Discussion

Ewers et al. reviewed 158 operations with successful use of IGS concluding that in the majority of cases, the medical benefit outweighed the technical expenditure [4]. Jeelani et al. utilized the StealthStation intraoperative navigation system to successfully perform a frontofacial monobloc distraction on a child with Apert syndrome [2]. Children with Pierre Robin Sequence, craniofacial microsomia, Treacher Collins, Crouzon (1 in 60,000), and Apert syndromes (1 in 65,000) can gain great functional and aesthetic benefits after such procedures [5, 6].

We describe our experience relative to 1) perceptions and 2) expenditures of IGS for this one patient. It is our perception that IGS, while not necessary for most routine craniomaxillofacial surgery is useful 1) as a teaching tool in the OR to highlight anatomy and 2) as an adjunct in cases of unusual anatomy, revision surgery, or complicated reconstructions. There is little or no data in the craniofacial literature in terms of improved safety, outcomes, patient aesthetics, or decreased need for further surgery. Our perceptions in terms of potential downsides of IGS include increased time in the OR for setup and small risks of mounting fiducial marker in the case of infrared IGS systems. There are additional costs including those needed for preparation and performance of surgical procedures supported by IGS. The FUSION™ ENT on Medtronic StealthStation S7 Surgical Navigation System costs $359,000 and this as well as the $18,900 for the supplemental instrument set, and $1,076 for the new surgeon wireless mouse are fixed costs though in our case these were on trial by the hospital. The recurring costs include the StealthStation^®^ Spheres, which come 12 in a pack for $20 per sphere and any additional anesthesia costs ($482/h) during the setup and calibration of the IGS. The total recurring costs were $481.

Recurring costs that are not part of the IGS system include the cost of the rigid external distraction (RED) system (KLS Martin, Jacksonville, FL) and the stereolithographic model (SLM), which totaled $14,500. The hospital stay accrued to $33,144 in the pediatric intensive care unit and $8,384 on the pediatric floor. The sum of these expenditures ($56,028) far exceeds the recurring costs of the IGS. The financial benefit of improved clinical outcomes or decreased complications is unknown at this point—ideally randomized case controlled studies would help in answering such questions although it would be impossible to blind such studies.

## Conclusions

We demonstrate that IGS can be easily integrated into clinical use with only 30 min of added time to the case, although further studies are certainly warranted to discover if IGS leads to decreased lengths of stay, reduced return trips to the operating room, and/or reduced complications. While the costs of IGS are not inconsequential, most of these costs are up-front and the recurring costs are relatively minimal when compared with the total costs of caring for children with complex craniofacial deformities including the need for multiple surgeries, at times lengthy hospital admissions, as well as the need for expensive hardware, devices, and diagnostic imaging tests. IGS allows junior craniofacial and plastic surgeons, oral surgery or otolaryngology residents, fellows, or medical students with an interest in craniofacial surgery the opportunity to visualize otherwise unseen anatomy using multi-planar radiographic data points [7]. Syndromic patients requiring craniofacial surgery often have unusual and/or asymmetric anatomy that is difficult to visualize or conceptualize by the novice surgeon. IGS in craniofacial surgery has the potential to allow for better teaching of difficult to conceptualize three-dimensional anatomy, which is often distorted by disease, and may lead to better understanding and mastery of complex craniofacial surgery.

## Consent

Written informed consent was obtained from the patient for the publication of this report and accompanying images.
